# Continuous nursing care improving outcomes of patients after percutaneous coronary intervention

**DOI:** 10.1097/MD.0000000000040807

**Published:** 2024-11-29

**Authors:** Yuan-yuan Zhang, Li-Xia Cui, Ling Zhang, You Wang

**Affiliations:** a Department of Cardiology, The Hospital of 82nd Group Army PLA, Baoding, Hebei Province, China; b Department of Cardiology, Nanjing BenQ Medical Center, The Affiliated BenQ Hospital of Nanjing Medical University, Nanjing, Jiangsu Province, China.

**Keywords:** continuous nursing care, coronary heart disease, percutaneous coronary intervention, WeChat

## Abstract

Percutaneous coronary intervention (PCI) is an effective method for the treatment of coronary heart disease. Cardiac rehabilitation and continuous nursing management after discharge are the key to ensure the quality of treatment and the quality of life of patients. This study aimed to explore the significance of WeChat platform in continuous care after PCI. We collected the clinical data of 90 PCI patients in 2 centers from January 2020 to July 2023, and divided the patients into 2 groups: the continuous nursing care group based on the WeChat platform and the control group. Continuous nursing care was implemented immediately after discharge. After 1-year follow-up, we compared the outcomes of the 2 groups, including postoperative complications, lifestyle changes and psychological status. There were no heart-related postoperative complications in either group. The incidence of vagal reflex was significantly decreased in the continuous nursing group (*P* = .042). The improvement of life style, such as smoking cessation (*P* = .001) and alcohol withdrawal (*P* = .003), was significantly better than that of the control group. The continuous nursing group was better than the control group in improving psychological status based on the Hamilton’s depression scale. Continuous nursing care based on WeChat platform could reduce the incidence of complications of patients after PCI, alleviate patients’ depression, and improve patients’ quality of life.

## 1. Introduction

Coronary artery disease is the leading cause of death worldwide, and its main pathological feature is myocardial infarction or ischemic cardiomyopathy caused by atherosclerosis.^[1]^ Due to its clear advantages in reducing mortality and myocardial infarction, percutaneous coronary intervention (PCI) in the form of balloon angioplasty and/or stent implantation has been increasingly used in the initial management strategy.^[2,3]^ However, the risk factors inducing atherosclerosis are not eliminated by PCI. To reduce the risk of reoccurrence, long-term pharmacotherapy is still needed after hospital discharge. Due to lack of related knowledge and poor self-management after discharge, the PCI patients surge psychological burden and deteriorate their quality of life.^[4]^ Continuity of care following PCI may play a key role in improving quality of life and mental well-being.^[5]^ Continuous nursing has been widely used in the management of chronic disease patients and has achieved satisfactory clinical results.^[6]^ As a rising star of the past decade, telemedicine has been centralized the most of the evaluated innovative interventions. Telemedicine is an expanded definition to include procedures that transmit medical information to provide or improve general well-being of patients, and has been shown to be helpful for patients’ telerehabilitation education and have a remarkable effect to increase patient’s eHealth literacy skills.^[7]^ Telemedicine technologies will be easily accessible via smartphones. The smart device and mobile application technologies have increased VO_2_ measurements and improved heart rate variability parameters in patients with high cardiovascular risk compared to conventional treatment alone.^[8,9]^

The main objective of this study was to investigate whether continuous care based on the WeChat platform can improve the outcomes of patients after percutaneous coronary intervention.

## 2. Materials and methods

This study was approved by our Ethics Committees, and all patients signed a written informed consent form.

### 2.1. Participants

We included 90 patients who met the inclusion and exclusion criteria from January 2020 to July 2023. Forty-six of them were placed into continuous nursing group, and the other 44 patients were placed into control group.

We included patients according to the following inclusion criteria: adult patients were definitively diagnosed as coronary heart disease by the World Health Organization criteria, patients underwent PCI therapy, the residual stenosis after PCI was <30% shown by coronary angiography, the follow-up duration was no <1 year, the Hamilton’s depression scale was completed by all patients at hospital discharge and during follow up after discharge.

The initially included patients were excluded after evaluation by the exclusion criteria: patients complicated with aortic stenosis, valvular disease, hypertrophic obstructive cardiomyopathy, tumors, severe hepatic or renal insuﬃciency, patients with postoperative complications, such as bleeding, embolism, restenosis, or occlusion, patients with loss of consciousness, mental illness, physical dysfunction, disability, or who were unable to cooperate with rehabilitation, patients who were lack of communication devices.

### 2.2. Nursing intervention procedure

Control group: During the period of hospitalization, nurses provided routine medical care, including dietary guidance, preoperative preparation, postoperative care, and related health education. Routine follow-up was given after discharge.

Continuous nursing group: Patients received conventional nursing care during their hospitalization. The continuous nursing care was provided by using WeChat platform at discharge, and the nurses instructed patients to use the WeChat function correctly and carefully. The length of intervention was 1 year after discharge.

WeChat health education included rehabilitation, personal health, and WeChat support. The rehabilitation included postoperative diet, exercise, drug therapy, daily behavior observation, postoperative psychological counseling and other rehabilitation related information, including smoking cessation and limiting alcohol. The personal health featured the patient’s main health profile and contains information on daily eating habits, sleep habits, exercise, mental state, medication reminders, and more. The online WeChat support was provided by our team from 7 to 10 pm every day to provide patients with possible help.^[10]^

### 2.3. Statistic analysis

We used SPSS version 16.0 to analyze continuous data by the *t* test or Mann–Whitney *U* test, and discontinuous data by the chi-square test or Fisher’s test. The *P* < .05 was considered as statistically significant.

## 3. Results

There were 46 and 44 patients in the continuous nursing group and control group, respectively. The statistical differences between the 2 groups were not significant in terms of age, gender, hypertension, diabetes mellitus, hyperlipemia, smoking, drinking, coronary heart disease (Table 1), or Hamilton’s depression scale (Table 2).

**Table 1:** Base-line characteristics of patients.

Item	Continuous nursing group	Control group	*P* value
Cases (N)	46	44	
Age (yr)	63.2 ± 11.3	63.4 ± 12.0	.960
Female/male	13/33	11/33	.727
Hypertension	37	32	.387
Diabetes mellitus	18	16	.787
Hyperlipemia	10	6	.315
Smoking	33	33	.727
Drinking	22	24	.524
Coronary heart disease			.204
1-vessel disease	18	19	
2-vessel disease	8	13	
3-vessel disease	20	12	

**Table 2:** Effects of continuous nursing on patients at 1-year follow up after percutaneous coronary intervention.

Item	Continuous nursing group	Control group	*P* value
Vagus reflex	1	6	.042
Smoking cessation	33	12	.001
Drinking cessation	22	8	.003
HADS scores			
Before PCI	8.6 ± 5.3	7.4 ± 4.8	.257
After PCI	0.9 ± 1.2	4.5 ± 3.5	<.001
Scores reduction	7.7 ± 4.5	3.0 ± 2.16	<.001

HADS = Hamilton’s Depression Scale, PCI = Percutaneous Coronary Intervention.

The postoperative heart-related complications, including hematoma, arrhythmia, coronary perforation, pericardial effusion, stent thrombosis, myocardial infarction, or death, were not found during the follow-up duration. The incidence of vagal reflex was significantly decreased in the continuous nursing group (*P* = .042). The smoking cessation (*P* = .001) and alcohol withdrawal (*P* = .003) were significantly better in the continuous nursing group than that in the control group. The continuous nursing group was better than the control group in improving depression based on the Hamilton’s depression scale (Table 2).

## 4. Discussion

Percutaneous coronary intervention (PCI) is increasingly used in the treatment of coronary artery disease, but it also increases the management of patients after PCI. With increasing patient health education and engagement, nursing plays a key role in upgrading healthcare delivery and improving patient satisfaction, mainly in terms of reducing disease risk, improving mental health and improving quality of life.^[5]^

In the current study, we found that continuous nursing care could benefit patients significantly in risk reduction (reduced vagal reflex), psychological improvement (reduced Hamilton’s depression scale), and quality of life (smoking cessation and alcohol cessation).

In order to strictly control blood pressure, blood glucose and blood lipid levels after PCI, patients should adhere to a healthy lifestyle and long-term medication. Some patients may experience a heavy psychological burden after PCI, especially those living in remote rural areas. Because these patients have a relatively low level of education and lack of relevant medical knowledge, they do not learn how to manage their disease to improve their health during the short hospital stay after PCI. As a result, these patients are prone to psychological problems such as depression and anxiety after discharge.^[11]^ However, with the popularity of the WeChat platform, these patients have the opportunity to receive continuous medical education and guidance.^[10,12]^ Due to the visibility and timeliness of the WeChat platform, patients can quickly solve their sudden problems with the remote help of the medical team through the special WeChat platform. Our team members take turns to provide online help to patients on the WeChat platform from 7 to 10 pm every day. At the same time, through the WeChat platform, we can also quickly assess the psychological state of patients and timely alleviate the negative emotions of patients.

The continuous nursing care through the WeChat platform has the following advantages. First, the medical staff can timely understand and solve the problems of patients, improve the standardization and effectiveness of cardiac rehabilitation management, and save a lot of time and economic costs for patients. Secondly, the forms of information sharing on the WeChat platform are diversified, including text, voice, animation, video, etc, which is conducive to patients with different educational levels to choose their own information expression methods to promote 2-way communication of information, so as to ensure the accuracy and professionalism of nursing intervention, and at the same time facilitate patients’ understanding and acceptance. Finally, patients can also communicate with each other through the WeChat groups to share their own experiences, so as to improve patients’ enthusiasm for self-management and promote the effect of long-term rehabilitation management.

Telemedicine is described as a medical approach that uses digital systems to guide patients to improve their health. For example, the use of audio and video to track the daily activities of patients to achieve personalized assessment of patient health status and targeted health care.^[7]^ In telemedicine, the implementation of cardiac rehabilitation portal is especially helpful to the remote rehabilitation education of PCI patients, and has a significant effect on improving the rehabilitation of patients.^[13]^ Telemedicine has made significant advances in cardiology over the past decade, but there are still inevitable barriers between data access, clinical trial registration, and new telemedicine technologies entering the public domain. However, with the popularity of smart phones equipped with multiple information exchange modes, such as the WeChat platform, telemedicine will become a milestone in the future of cardiac rehabilitation.^[14]^

The retrospective analysis with a relatively small sample size is a main limitation of this study. A multi-center prospective study with randomized double-blind design is needed in the future.

In conclusion, continuous nursing care through the WeChat platform can extend high-quality medical care and psychological support to patients after PCI.

## Author contributions

**Conceptualization:** Yuan-yuan Zhang, You Wang.

**Data curation:** Yuan-yuan Zhang, Li-Xia Cui.

**Methodology:** Yuan-yuan Zhang, Li-Xia Cui, Ling Zhang, You Wang.

**Supervision:** Ling Zhang.

**Writing – original draft:** Yuan-yuan Zhang.

**Writing – review & editing:** Ling Zhang, You Wang.
